# Hospital Meetings

**Published:** 1905-04-01

**Authors:** 


### HOSPITAL MEETINGS.

### ROYAL FREE HOSPITAL.

Princess Christian, who was accompanied by Princess Louise Augusta of Schleswig-Holstein, presided at the 77th Annual Court of Governors, at the Royal Free Hospital, Gray's Inn Road. Among those present were Lord Sandwich (Chairman of the Committee of Management), Lady Reay, Lady Llangattock, Mr. Charles Burt (Chairman of the Weekly Board), Major Ricardo, Mr. Thomas Wakley, and the secretary (Mr. C. W. Thies).

Lord Sandwich moved the adoption of the annual report, and said that the committee had much pleasure in announcing that Queen Alexandra, patron of the hospital, had again shown her gracious and practical interest in its work by making a liberal contribution towards the cost of the structural and other improvements in the hospital buildings carried out during the past year. These included improvements to the Sussex and Victoria wing wards, and additions to the nurses' quarters, including the provision of an electric lift for the Victoria wing staircase.

Mr. Charles Burt, chairman of the Weekly Board, in seconding the adoption of the report, said that during the past year 2,364 in-patients, 41,701 out-patients and casualties, and 349 maternity cases were treated.

The report and financial statement having been adopted, the committee of management was appointed. The thanks of the court were given to Princess Louise Augusta of Schleswig-Holstein, president of the Ladies' Association, and to the members of the Association for the help given to the hospital during the year. Presentations were made to Miss Wedgwood, the late matron, who, after 13 years' service, is being succeeded by Miss Cox-Davies, and to Mr. A. Boyce Barrow, the late senior surgeon.

Princess Christian, in reply to a vote of thanks for presiding, said she was very grateful to them all, and before they separated she would like to add her word of sorrow and regret for the loss of their matron, Miss Wedgwood, who was an old and valued friend of hers. They would miss her very much.

Lord Sandwich said that Princess Christian had given her sanction to inscriptions on tablets in the nurses' new room and on a bed in Milne Ward in her honour, and on a bed in Marsden Ward in memory of the late Prince Christian Victor, who died in Pretoria on October 29th, 1900.

Princess Christian visited the Victoria wing, where she inaugurated the new electric lift and other improvements, and, with Princess Louise Augusta, remained to tea.

### NATIONAL HOSPITAL FOR THE PARALYSED AND EPILEPTIC.

The annual general meeting of the Governors of the National Hospital for the Paralysed was held on March 21st, H.R.H. the Duchess of Albany being present, the chairman, Mr. J. Danvers Power, who is about to retire, presiding for the last time.

Mr. Power pointed out the large sum that had been saved to the hospital in various ways during last year, but especially he desired to thank the committee of ladies, who had worked so successfully that their labours resulted in the saving of nearly £1,700 per year in housekeeping.

A considerable reduction in the drug bill had taken place, and the medical staff worked heart and soul with the board of management to keep down expenses without deleterious results to the inmates.

Mr. Danvers Power was of opinion that it was chiefly in housekeeping, however, that they must look for results in economising, and he congratulated the hospital on securing the services of Miss Daunt as housekeeper, instead of, as heretofore, having a steward. In spite, however, of all that had been gained by improved management the hospital was in a bankrupt condition—at least another £5,000 a year was needed to carry on the institution satisfactorily.

By the kindness of Mr. Beerbohm Tree and many members of the dramatic profession a matinée was given in March, which brought to the hospital the sum of £1,290.

These and other handsome sums have helped to tide over difficulties, but it has been unfortunately found necessary to close the Convalescent Home to all but paying patients. He hoped that the public would come forward generously to support a charity that was of world-wide interest.

Sir James Crichton-Browne seconded the motion for carrying the report.

Mr. MacMillan moved the adoption of the accounts, and reproached Mr. Danvers Power for saying they were in a bankrupt condition, which, were they engaged in a trading concern, would certainly be a true description, but as hitherto they had never appealed to the subscribing public without a good result, he hoped that they might still rely upon being generously supported.

Dr. Buzzard moved the resolution to appoint Lord Dudley president; Dr. Ormerod seconded the motion, which was carried.

The proceedings closed by H.R.H. the Duchess of Albany thanking Mr. Danvers Power for his close attention to the affairs of the hospital during his term of office, and expressing her gratification at the improved condition of the hospital in all respects.

### THE LEEDS INFIRMARY.

Mr. Lupton, the Treasurer, made an interesting speech at the annual meeting of the Leeds General Infirmary this week. He stated that there had been an increase in the amount of work done in every department, and that the average residence of each patient was 18·7 days, a shorter period than ever before. Despite the increase of £955 in the income and an increase of £834 in the expenditure for the year, the receipts exceeded the expenditure by upwards of £1,000. Referring to the speech made by the Prince of Wales on Hospital Economy, Mr. Lupton stated that Sir Henry Burdett had promised to look through the figures of the Leeds Infirmary and see if he could make any suggestions with regard to the management. The plan followed by the National Hospital for the Paralysed in the direction of domestic economy had been in use at the Leeds Infirmary for years. They were beginning to feel the cost of an oldish building which entailed large expenditure on repairs Mr. Lupton was opposed to the levy of compulsory payment. upon hospital patients, for there was a feeling growing up amongst the working classes that those who could afford to pay should go to their own doctor. The infirmary has to depend upon benefactions and legacies to the extent of £12,000 a year.

### QUEEN'S JUBILEE HOSPITAL.

### The Resignation of the Medical Staff.

At the annual meeting of this hospital Mr. Hayes Fisher, M.P., presided. Mr. Everett, the Chairman of the Board, reported that it had been decided to lay all the facts in connection with the resignation of the Medical Staff before the King's Hospital Fund in the form of a report, which he read. This report was published in full in the *Times* of the 27th inst., and set forth the facts from the committee's point of view. Pending any action which the King's Fund may take, we have decided not to publish the report, and in any case it would occupy more space than we can afford this week. The annual report was adopted on the motion of Mr. Hayes Fisher, who stated that he had great confidence in the hospital, that there was nothing seriously wrong, nothing that could not be set right. He has subsequently stated that the real need of the hospital is more money, and such additions to its committee of management as will obtain for it the full confidence of the charitable public. From his intimate acquaintance of the working classes of Fulham Mr. Hayes Fisher writes that, notwithstanding its inadequate accommodation and resources, the poor people of the district resort to the Queen's Jubilee Hospital, and invariably speak well of the treatment accorded to them.

### THE NATIONAL ASSOCIATION FOR THE PREVENTION OF CONSUMPTION.

In the annual report of the above association it is announced that a meeting which will bring the claims of the association before the City will be held at the Mansion House, under the presidency of the Lord Mayor, on May 17th, and the next International Tuberculosis Congress will take place in Paris, under the patronage of the French President, from October 2nd to 7th. The aid of the association has been sought by the authorities of the congress to co-operate with them in this country, and it is hoped that the meeting of the congress will give a fresh impetus to the efforts of the association and rouse new interest in the questions of tuberculosis. Tuberculosis in children, especially the surgical forms of the disease, has engaged the attention of the association during the past year, and a sub-committee have made recommendations urging the necessity for the provision of special hospitals for these cases, in the country or at the seaside, on the lines of open-air sanatoria for pulmonary consumption. For the incurable cases separate homes should be provided. A gratifying step in the extension of public control with regard to tuberculosis has been taken by the Sandgate Urban District Council, who are seeking power, *inter alia*, to exercise control over hospitals or sanatoria for tuberculosis, to introduce notification of the disease, and to regulate the milk supply. The council say that the time is evidently close at hand when provision of sanatoria, municipal and other, will be on the increase. Some system, too, must be devised to spread information as to the initial symptoms of phthisis and the importance of really early treatment. In all this there is a wide field for private endeavour to supplement public action.

### ROYAL NATIONAL HOSPITAL FOR CONSUMPTION, VENTNOR.

Lord Rosebery, in moving the adoption of the report and accounts of this hospital on the 23rd ult., referred to the great success of the method adopted for reducing the waiting list—*i.e.* the raising of the qualification from three to five guineas, and the issue of letters only on the application of Governors. There were now only 84 patients awaiting admission, as compared with 239 before the rule came into force. He was glad to say that the Governors had given it their cordial support.

The deputy-chairman, Mr. G. Thompson Powell, gave some details of the year's work, showing that 969 patients had been treated, 41 of whom were admitted free. The average stay was 66·465 days, and there were 817 completed cases. The expenditure exceeded the receipts by £2,961, and the hospital owed £3,000 to the bankers. In reliance on special efforts being made to meet this loan and the necessary expenditure for 1905, the board were keeping all the beds fully occupied. Taking into account the excellence of the patients' food and the fact that each had a separate room, he thought the average cost of £100 per bed per annum was not excessive.

The Board regretted that although a large percentage of the cases came from London and the neighbourhood, the Hospital did not benefit either from King Edward's or the Hospital Saturday Fund; they wished to emphasize this, as many large contributors to these Funds now limited their subscriptions to them.

The report and accounts having been passed, Dr. Robertson gave some interesting statistics covering a period of 10 years, showing improvement in every respect, more especially in the percentage of improved cases (males 100 per cent., females 94·3 per cent.). He strongly urged the Governors to grant letters only to applicants who appeared suitable on a medical opinion, advanced cases being expensive and benefiting little.

### MOUNT VERNON HOSPITAL FOR CONSUMPTION AND DISEASES OF THE CHEST.

The annual Court of Governors of Mount Vernon Hospital was held at the Out-patient Department, in Fitzroy Square, Mr. J. S. Fletcher being in the chair.

He began by saying how 1904 would always be a memorable year to them, for it saw the completion of the hospital, which has been of slow growth; it was 40 years since the institution was started, and then in a very small way. The central Out-patients' Department was now in full working order, and the country hospital at Northwood was completed and opened with a few patients. He went on to speak of the financial position of the charity, which, like most others of the same kind, was in a bad way, and at present it is impossible to open more than 140 beds. This is a grievous state of things, considering how urgent and constant are the demands for admission, and that there remain 110 beds lying idle, but until more money is forthcoming it is impossible to incur further monetary responsibilities.

Mr. Fletcher remarked that the novelty of open-air treatment having passed, the first flush of excitement, leading to liberal donations and subscriptions, has passed too, with regard to new supporters, but it is gratifying to know that old and constant subscribers still continue their help. More money must, however, be got, and the committee have decided to appeal to the public for the large sum of £100,000.

It was gratifying, Mr. Fletcher said, to note the preventive work achieved by the Out-patients' Department. The sufferers learn the value and importance of personal cleanliness, the danger to the community of spitting, and the absolute necessity of fresh air. Moreover, all applicants for admission are examined in Fitzroy Square, and every relief possible is afforded to those who have often, unfortunately, to wait a weary time before they can be received at Hampstead.

A new rule was passed by which a sum of 50 guineas instead of 30 guineas was necessary to constitute a life governorship, and that 5 guineas instead of 3 guineas should be the sum paid by an ordinary governor, who has the privilege of recommending one in- and four out-patients annually. These alterations only apply to governors and life governors who have become so since March 1905.

Another rule, by the wish of the medical staff, has been made that no physician or surgeon shall absent himself from his duties for more than a week at a time without giving notice to and obtaining the leave of the committee, and providing a suitable substitute of equal medical qualifications, whose name shall be notified to and approved by the committee.

### ST. MARY'S HOSPITAL.

The annual meeting of Governors of St. Mary's Hospital, Paddington, was held in the Board-room on March 24th, under the chairmanship of Mr. Harbin. The income of the charity was less by £10,815 than in 1903, except that a sum of £7,000 was received for the exclusive benefit of the Medical School, so that it could not be used for the general maintenance fund.

Of this large falling off, £3,250 is in endowment donations, nothing having been received for this purpose in 1904. The remainder is in legacies, which came to £14,309 in 1903 and n 1904 to £7,095 only. The consequence is a deficiency at the end of the year of £9,918.

For many years the annual expenditure has amounted in round figures to £30,000, and the ordinary income, exclusive of legacies, has fallen short of £15,000. Surgery and dispensary expenses are the only heads of expenditure under which St. Mary's does not compare favourably with other similar charities. This unwelcome fact attracted the attention of the Board during the year, and it is receiving careful consideration.

Regret was expressed at the death of Mr. Alfred Scorer, of Mr. A. Q. Silcock, and the Rev. Walter Abbott, vicar of Paddington.

### ST. PETER'S HOSPITAL.

Mr. Edwin Fox, presiding at the annual meeting of St. Peter's Hospital for Stone and Genito-Urinary Diseases said that in order to provide for a greater number of patients in future the committee were seriously contemplating placing six beds for patients able to pay moderate fees in the present board room. The increase in the attendance of medical men (from 1,141 to 1,186) indicated how greatly the work in the particular branch of surgery practised was appreciated. During the year electric light had been carried to the wards. The receipts from donations and subscriptions were not so large as in the past two years, and had it not been for legacies amounting to £1,700, the deficit on the year's working would have been about £600. Amongst items of expenditure he drew attention o rates and taxes, from which he was strongly of opinion the voluntary hospitals should be relieved. The grants from the Central Funds were larger than ever before, amounting to £228 16s. 8d.

Mr. F. Swinford Edwards, in presenting the surgical report, said the high death-rate during 1904 (32 in 449 admitted), when analysed, showed that 13 patients died without any operation, while others were admitted at too advanced a stage to render operation successful. The number of serious cases also accounted for the falling off of 107 patients from the number admitted in the previous year.

Both reports having been adopted, the names of Sir George Livesey, Mr. H. Samuel, and Captain Sloane Stanley were added to the committee, and the proceedings terminated with the usual votes of thanks.

